# Community Social Cohesion During a Large Public Housing and Neighborhood Redevelopment: A Mixed Methods Study

**DOI:** 10.3390/soc15050140

**Published:** 2025-05-20

**Authors:** Judith L. Perrigo, Anna Ginther, Haniya S. Syeda, Victoria Shier, Ashlesha Datar

**Affiliations:** 1 Luskin School of Public Affairs Social Welfare Department, University of California Los Angeles (UCLA), 337 Charles E Young Dr E, Los Angeles, CA 90095, USA; 2 Schaeffer Center for Health Policy & Economics, University of Southern California (USC), Los Angeles, CA 90089, USA; 3 Center for Economic and Social Research, University of Southern California (USC), Los Angeles, CA 90089, USA

**Keywords:** social cohesion, public housing redevelopment

## Abstract

Public housing redevelopments can disrupt community members’ sense of belonging and attachment to their neighborhood, in large part due to resident displacement and gentrification. Recent public housing redevelopment efforts seek to mitigate these adverse changes, but evidence of their association with community social cohesion is limited. The current mixed methods study examines community social cohesion from different perspectives during a large public housing and neighborhood redevelopment in Southern California. Semi-structured qualitative interviews (*n* = 21) were conducted with various community stakeholders to explore their perceptions of social cohesion within the context of public housing and neighborhood redevelopment. Additionally, the Social Cohesion scale was used to evaluate residents’ (*n* = 647) sense of neighborhood social cohesion. Stakeholders raised concerns about how redevelopment could disrupt the social fabric of their communities and underscored the importance of deliberate efforts to promote integration between existing and incoming residents. The significance of creating physical spaces, protecting landmarks, and facilitating social interaction to cultivate a sense of belonging was also emphasized. Residents of the public housing redevelopment reported significantly higher levels of social cohesion when compared to residents from two neighboring public housing complexes that are not undergoing redevelopment. Implications for public housing redevelopment strategies, community integration efforts, and future research are discussed.

## 1. Introduction

Housing plays a critical role in shaping the health, wellbeing, and stability of individuals and communities. In addition to providing physical shelter, housing has been implicated in a range of social and psychological processes, including socialization and community formation [1]. This broader recognition of housing’s association with social and psychological wellbeing underlies the importance of increased governmental involvement in ensuring access to adequate housing for vulnerable populations. Consequently, the United States federal government has played a major role in housing assistance, including the provision of public housing for low-income families since the 1930s. By the early 1990s, however, public housing became a symbol of concentrated poverty, high crime, and distressed housing, leading to the implementation of the Housing Opportunities for People Everywhere (HOPE VI) program [2,3]. HOPE VI granted over USD 6 billion in grants for deconcentrating poverty and redeveloping distressed public housing into mixed-income developments. The HOPE VI program was associated with mixed success [4], leading to its replacement in 2010 by the Choice Neighborhoods Initiative (CNI). Since its implementation, the CNI has awarded over USD 1 billion to support low-income housing redevelopment with a continued emphasis on producing mixed-income housing. But, unlike HOPE-VI, CNI emphasizes focusing beyond redeveloping the distressed public housing sites to complete neighborhood transformation, community engagement in the planning and implementation of the redevelopments, and improvement in residents’ outcomes.

While these redevelopments promise improved infrastructure, economic growth, and tenant protections, they can also have profound social implications for neighborhood residents. For instance, changes in neighborhoods can significantly impact resident’s attachment to their neighborhood, in terms of both personal and collective identity, as well as their sense of belonging. Neighborhood disruptions, therefore, have the potential to impact the identities of neighborhoods, which fuel a sense of community.

### 1.1. Theoretical Framing

Social cohesion has garnered increasing attention in academic and political fields, and is often conceptualized through the complex interplay of both individual-level (e.g., attitudes, behaviors) and group-level (e.g., shared identity) factors [5,6]. Conceptualizations of social cohesion vary across disciplines, emphasizing different characteristics such as multiculturalism, conflict, and social capital [7]. Additionally, definitions of social cohesion have shifted with sociopolitical ideologies [6]. For example, concerns about a perceived lack of social cohesion within disadvantaged neighborhoods have emerged in relation to urbanization, implicitly centering the shared values and norms of more privileged communities in describing social cohesion [8,9]. However, key elements of social cohesion across definitions have been identified, including social relations, belonging, and shared values [6,7,9].

The built environment plays an important role in shaping community social cohesion—defined in this study as the sense of belonging within a community and the nature of relationships as perceived by community members [10–12]. Our understanding of how public housing and neighborhood redevelopment may influence community social cohesion and residents’ connection to the places they inhabit can be informed by place identity theory (PIT). The theory, developed by Proshanky et al. [13], posits that place identity comprises various cognitions such as memories, ideas, feelings, meanings, and values that are centered around the individual’s physical, built environment. These cognitions are deeply rooted in the person’s environmental past, including places, spaces, and properties that have fulfilled their biological, psychological, social, and cultural needs. In essence, an individual’s sense of self is partly defined by their relationships with the physical settings in their daily life. This includes, but is not limited to, local neighborhood parks, shops, restaurants, and urban monumental sights.

Neighborhood continuity is a crucial aspect of maintaining a sense of self-identity to place [13]. For instance, an individual’s recognition of community characteristics provides tangible reminders of their past, forming bridges to the present and fostering a sense of continuity. This ongoing recognition of the neighborhood strengthens place identity, nurtures attachment to the environment, and enhances one’s overall sense of belonging. Consequently, changes in the physical environment—such as those that occur during redevelopment can threaten this sense of place identity and social cohesion.

### 1.2. Background

Prior research supports this theory, showing that the built environment is associated with residents’ sense of belonging and harmony with their physical and social surroundings [14,15]. Belongingness can also include a sense of ownership and entitlement to a particular space, conveyed through the physical and visual cues embedded in a place’s architecture, development, and spatial organization [16]. In this way, belongingness functions as a process of claiming ownership of a place, like signaling that a particular group belongs there [16]. Public housing redevelopment can potentially disrupt this sense of claim and impact the overall feeling of belongingness within a community.

Changes in spatial characteristics attributed to redevelopment may also lead to the social exclusion of long-term neighborhood residents. For instance, a study found that aging residents witnessed the transformation of their neighborhood; new consumption spaces like wine bars, coffee shops, and vegetarian cafes emerged, catering to a younger demographic [17]. Many long-term residents expressed concerns that the familiar built environment and close-knit relationships defining their community were being threatened by the contrasting lifestyle the space was now promoting. In another example, White [16] describes how a neighborhood redevelopment project disrupted a key cultural element in a community. Specifically, a group of musicians gathered in a local park for over 30 years but were suddenly disrupted by police officers responding to noise complaints from new residents [16].

This incident interfered with a long-standing cultural practice and prompted inquiries into the ownership and claim of the public space, which had been a cornerstone of cultural expression. This practice not only embodied the community’s cultural and place identity but also acted as a gathering space that nurtured long-standing social connections and a sense of belongingness. Though the redevelopment did not physically displace residents, this example illustrates how such projects can cause psychological displacement, as residents may no longer freely express their cultural identity. Even minor changes to physical spaces can disrupt the community fabric, leading to a loss of belonging despite continued physical presence.

In the United States, redevelopment efforts have differentially impacted social ties and cohesion in targeted communities. A qualitative study of residents and stakeholders in a community in Georgia found that gentrification exacerbated social fragmentation across a variety of demographic categories (e.g., residential tenure, race, age, income), thus limiting the sense of community social cohesion [18]. On the other hand, Farquhar and colleagues [19] observed a significant increase in reported social cohesion among residents of a redeveloping publicly subsidized housing community in Seattle. Another study of residents within a public housing redevelopment site found that concerns around social cohesion in the face of relocation varied; while some residents shared strong neighborhood social connections that could be disrupted by a move, other residents did not anticipate any significant impact on their social ties [20].

### 1.3. Current Study

The goals of the present study were to examine how community stakeholders perceive social cohesion during a large public housing and neighborhood redevelopment, and whether these perceptions align with residents’ experiences. To answer these questions, we conducted 21 semi-structured qualitative interviews with various community stakeholders, including residents, community entrepreneurs, law enforcement, and members from the housing authority. These interviews aimed to explore their perspectives on social cohesion during the early phases of a redevelopment project in Southern California. To determine if the qualitative findings were consistent with resident experiences, we then evaluated residents’ (*n* = 647) sense of neighborhood connectedness using the Social Cohesion scale [21]. By integrating qualitative and quantitative methods along with diverse community perspectives, we aimed to capture a comprehensive view of the complex social changes related to public housing and neighborhood redevelopment. Our study makes a meaningful contribution to the existing literature by: (1) integrating both qualitative and quantitative assessments of social cohesion within a single study; (2) incorporating multiple perspectives, including those of residents and broader stakeholders; and (3) examining a unique case in which residents remain in place during a public housing and neighborhood redevelopment—an occurrence that is relatively uncommon.

## 2. Methods

### 2.1. Study Setting

Jordan Downs is a public housing community in the Watts neighborhood in Los Angeles, California, that houses over 2300 low-income residents who are primarily Latino/a (67.7%) and Black/African American (31.1%) [22]. The Jordan Downs redevelopment is a public–private partnership led by the Housing Authority of the City of Los Angeles. The redevelopment is expected to cost over USD 1 billion, financed by private and public sources, including a federal CNI grant for some phases. Planning for the redevelopment began in 2008 and broke ground in 2017. Because Jordan Downs had an adjacent vacant parcel, the developers implemented a “build first” approach which avoids any involuntary displacement of residents during the redevelopment. It includes a one-to-one replacement of existing 700 units reserved for current residents, and an additional 869 units that will be constructed for new residents to gradually introduce mixed-income housing. Although the redevelopment is expected to be complete in 2029, almost one-third of original households of low-income residents moved into their new units by 2022, the year when the study data were collected. The new housing meets modern housing standards (i.e., LEED^®^ Gold Certification) and the neighborhood has a new supermarket with healthy food options. Additional neighborhood features include a large retail center with several national and local retailers, parks and green space, bike lanes, and pedestrian friendly streets with lighting and other safety features. In addition to redeveloping the Jordan Downs site, federal and state grants funded neighborhood-wide investments in Watts to increase community engagement, promote workforce development, and minimize resident displacement [23].

### 2.2. Study Participants

Study participants in the semi-structured qualitative interviews were selected by utilizing a purposive sampling strategy based on community members’ knowledge of ongoing improvements in Jordan Downs. While not generalizable, a purposive sample facilitates access to a wide range of community stakeholders that would otherwise be very difficult to reach. Overall, 25 potential community members were identified and invited to a semi-structured qualitative interview and 21 accepted, yielding a response rate of 84%. The reasons four community members did not participate include a lack of availability (*n* = 3) or not being interested in the study (*n* = 1). After completing informed consent procedures for participation in the study, each community member was provided with a brief survey (e.g., demographics) and then completed a 60 min semi-structured qualitative interview.

In addition to the qualitative semi-structured interviews, we analyzed survey data from residents collected as part of a larger cohort study underway—Watts Neighborhood Health Study (WNHS)—that is examining the effects of the redevelopment on residents’ health and wellbeing more broadly [24]. The WNHS recruited 888 adult residents from Jordan Downs and two other public housing sites in Watts between 2018 and 2020 and surveyed them annually. The participants are primarily (70%) Jordan Downs residents since it is the site of the redevelopment. The remaining participants are recruited from two other large public housing sites in Watts—Nickerson Gardens and Imperial Courts—that serve as a comparison group in the cohort study. The present study used data collected from *n* = 647 residents in the WNHS who were surveyed about their sense of social cohesion in their neighborhood between June 2021 and June 2022, which corresponds with the timing of the qualitative study.

Both the qualitative and quantitative research studies were approved by the Institutional Review Board at the affiliated university.

### 2.3. Data Collection

All interviews were conducted between July and August of 2021. Two research team members, including an academic faculty member and one trained graduate-level research, student completed all 21 interviews. Because of the COVID-19 pandemic, semi-structured interviews were conducted via Zoom and audio-recorded. Study participants were each offered a USD 100 gift card for their participation. This compensation was provided as a token of appreciation for their time, and in recognition of the participants’ invaluable contributions to the study.

Semi-structured interviews probed for community members’ opinions and sentiments related to the physical and social changes caused by the Jordan Downs redevelopment. Sample questions included the following: *Can you share your perspective and opinions on how you think mixed-income housing will or will not affect the community? What words or adjectives would you use to describe your current feelings about the Jordan Downs redevelopment and why?* Questions were purposely designed to be open-ended to provide community members with the opportunity to freely express their opinions. Each question was accompanied by probing questions to gain further insight.

Additionally, WNHS survey data were gathered from residents of Jordan Downs and two neighboring public housing sites, Imperial Courts and Nickerson Gardens, which served as comparison sites. These data were collected primarily through computer-assisted telephone interviews, with the option for in-person interviews if preferred by respondents [24]. Surveys collected information on participants’ socio-demographics (e.g., ethnoracial background, highest level of education, and age) as well as the length of time that they lived in their public housing site. In addition, a validated and extensively used five-item social cohesion instrument [21] asked participants to rate on a scale of one (strongly disagree) to five (strongly agree) how strongly they felt that people in their community are willing to help each other, can be trusted, get along with each other, and share the same values, and whether they believe their community is close-knit. Sampson et al.’s [21] scale was developed to explore how solidarity and mutual trust among neighbors interact with their willingness to intervene for the community’s benefit. The same response scale was also used to assess how strongly residents believed their community is a good place to live.

### 2.4. Data Analysis

Demographic characteristics, for both the qualitative and quantitative study participants, were subjected to descriptive statistical analysis, including averages and percentages.

#### Qualitative Data.

For the qualitative semi-structured interviews, audio recordings were professionally transcribed and reviewed by research team members to ensure accuracy. A codebook was created between three research team members, including one academic faculty member and two graduate-level research assistants. First, research team members open coded four randomly selected transcripts by taking the textual data and breaking it up into discrete parts. Blocks of text were coded and labeled with temporary code names during this phase. After the four transcripts were complete, the research team discussed axial coding to draw connections between the codes, identifying primary and secondary codes, and finalizing code names and definitions. In the final step, two research team members randomly selected two additional transcripts and independently coded the text using the codebook. Inter-rater reliability achieved high kappa scores of 0.96 for the primary level codes and 0.83 for the secondary level codes, indicating strong agreement between the researchers [25]. Once strong inter-rater reliability was established, the team coded the remaining 15 transcripts using the codebook. Deductive and inductive thematic coding were employed in this analysis [26]. Deductive codes were based on the interview questions, while inductive codes were based on the community members’ answers. To ensure consensus, any disagreements in coding were resolved by involving a third research team member. The coding of all 21 transcripts was conducted through a web-based qualitative data management program, as per Dedoose [27]. After all coding was complete, various coding trees were created to visualize code frequency. By performing this final step, researchers were able to see important central connections and assign weight to certain codes that captured the essence of community members’ perspectives and attitudes about the Jordan Downs redevelopment.

#### Quantitative Data.

Quantitative data were analyzed using the statistical software STATA 17 [28] using univariate and bivariate frequency distributions and bivariate cross-tabulations. All social cohesion questions were analyzed individually. In addition, a social cohesion index score was constructed adding up the scores on the 5 items that comprise this scale. We examined how responses to the social cohesion survey questions varied by whether the participants lived in Jordan Downs or the comparison public housing sites. Furthermore, since the redevelopment was only partially complete, we also divided the resident sample by whether they relocated to the newly redeveloped areas within Jordan Downs (New JD) or were still living in the yet-to-be-redeveloped areas (Old JD). This distinction is important because there was a growing dichotomy within Jordan Downs in terms of the physical conditions in the Old JD versus the New JD areas. In contrast to the newly redeveloped areas, there was greater physical disorder in the form of trash, graffiti, and boarded up or deteriorating buildings in Old JD, which may influence residents’ perceptions of their community. In addition, we also examined whether residents’ perceptions varied by length of residence due to prior positive associations between community well-being and housing tenure [29]. Given that redevelopment projects can potentially disrupt social connections, the authors proposed three hypotheses: (1) residents of Jordan Downs would exhibit lower levels of social cohesion compared to residents of the comparison sites, (2) residents of the New JD would demonstrate lower levels of social cohesion than those residing in the Old JD units, and (3) residents who lived longer in the community would have greater social cohesion relative to those who moved there more recently.

## 3. Results

### 3.1. Demographics

Table 1 presents the descriptive characteristics of community members who participated in the semi-structured qualitative interviews. In summary, the community members had an average age of 47 years, with a majority (62%) being female, approximately 52% of them held a master’s degree, and a third were residents of the Watts community. Slightly over eighty percent of our sample represented either Latino/a (43%) or Black/African American (38%) ethnoracial backgrounds. As for occupation, a significant portion of participants (57%) worked in the non-profit sector. On average, participants worked in their current organization for eight years.

Table 2 presents the descriptive characteristics of residents in the WNHS who completed a survey about their perceived social cohesion. Across both Jordans Downs and the comparison sites, the majority of respondents were female (New JD: 78%, Old JD: 73%, comparison: 75%) and not married (New JD: 76%, Old JD: 76%, comparison: 79%). The ethnoracial background of respondents in new and old Jordan Downs was primarily Latino/a (New JD: 75%, Old JD: 78%). In the comparison sites, 59% of respondents identified as Latino/a, while 41% identified as Black/African American. In all sites, approximately 80% of respondents were either aged between 18 and 34 (New JD: 40%, Old JD: 38%, comparison: 38%) or between 35 and 54 (New JD: 41%, Old JD: 37%, comparison: 43%). In old and new Jordan Downs, the largest proportion of respondents had a household income of USD 20,000 or higher (New JD: 40%, Old JD: 41%). On the other hand, nearly half of the respondents (48%) from the comparison sites had a household income of USD 9,999 or less. The greatest proportion of residents across Jordan Downs (New JD: 38%, Old JD: 42%) and comparison sites (comparison: 40%) had a high school or equivalent level of education.

### 3.2. Qualitative Themes

As stakeholders reflected on community social cohesion within the context of public housing and neighborhood redevelopment, two primary themes emerged. First, stakeholders underscored the importance of deliberate efforts to bridge the potential divide between existing and incoming residents, stressing the necessity of fostering integration among all community members. Second, they highlighted the significance of creating physical spaces, protecting landmarks, and facilitating opportunities for social interaction to cultivate a sense of belonging.

#### Bridging Residents for Community Integration.

Stakeholders described several potential disruptions to the social fabric of their communities, resulting from public housing and neighborhood redevelopment. Questions were raised regarding the potential effects of new residents’ arrival on existing relationships, and the broader implications for community social cohesion. One member stated the following:

There are adjustments in who your neighbors were and your connection to those previous neighbors. Then, there’s going to be the dynamic of new people who may not be from Watts that come into the community.

As more people, different populations come into the community, these aren’t going to be organic people from the community. It’s going to be people outside of the community. And so because of that, you know, the culture of the community changes. And it’s like, we have a strong culture that’s been pushed to the wayside.

In general, stakeholders were worried about the integration of new residents—who may not appreciate or understand the neighborhood culture—and how this might alter existing community dynamics. A community member shared, “*There has to be a way to bring folks in without compromising the fidelity or community integrity that makes Watts*,” indicating that the neighborhood is viewed as an essential space for community members to relate to one another. Another community member explained the following:

There’s not enough being done in terms of building social cohesion between the groups and understanding and fostering of neighborly co-existence. That’s not happening. That’s what it used to be, right? You used to have a neighborly co-existence where you could check up on each other’s kids as they’re walking to school and you knew your neighbor.

While stakeholders expressed the need for community integration in general, several specifically emphasized the critical importance of incorporating individuals from diverse ethnoracial backgrounds. The Watts community has undergone a significant shift in ethnoracial demographics, transitioning from a predominantly Black/African American population in the 20th century to a predominantly Latino/a population (77%) in 2022 [30]. Multiple perspectives highlighted the perceived state of the community, where racial tension was viewed as an obstacle to the successful integration of both new and existing residents. As one resident questioned, “*How do we continue to build community across race and ethnicity, and an understanding that it’s not helpful for folks to be divided in that way? So I do think some of that needs to happen*.”

Another dimension of community integration that was discussed included how the physical space impacts the social environment. Stakeholders shared how the public housing and neighborhood redevelopment disrupted previously established social interactions among existing neighbors. As one stakeholder described, unlike the newly redeveloped housing, “*the previous building at Jordan Downs had mini porches and lawns. You could see your neighbor just across your lawn*… *the new phases that have already been constructed; the building doesn’t lend itself as easily to that. I believe that has introduced a disruption to a certain cultural element or cohesion of the community*.” This sentiment underscores stakeholders’ perception that the built environment plays a crucial role in facilitating social interactions, and that public housing redevelopment can disrupt this dynamic.

Collectively, these perspectives suggest apprehension about change within the community. In light of the public housing redevelopment, stakeholders emphasized the importance of maintaining elements of familiarity to support the integration of new residents and to preserve the connection between individuals and their physical spaces. Overall, stakeholders expressed concerns that the influx of new residents could lead to disruptions in the social fabric of the community, stressing the need for enhanced efforts to promote community integration.

#### Cultivating Spaces for Social Belonging.

Stakeholders shared that a sense of community belonging extends beyond just the physical space, embodying a deeply rooted connection to a shared built and social environment. Division can be created, stakeholders argued, if residents are not given the opportunity to acclimate to their redeveloped housing. One stakeholder somberly stated, “*With moving to the new development, we don’t feel like we belong*.”

The lack of belonging that stakeholders described was multifaceted, including new surveillance from the housing authority, the potential removal of historical landmarks, and overall changes in the built environment. For instance, one stakeholder described the social dynamics with the new management, stating that “*Especially with the management* … *They watch us all the time, that we can’t do certain things. The kids step out to play a little bit* … *it’s not good. They don’t want them to be playing outside. They question them. ‘What are you doing here?’ You know, things like that*.”

Stakeholders underscored the critical importance of cultivating a sense of belonging as a foundation for establishing collective identity and solidarity within their increasingly diverse community. Additionally, they highlighted the necessity of shaping the social fabric of newly redeveloped spaces. Concerns were raised regarding the potential threat redevelopment poses to community member’s connections to the land. One stakeholder, in particular, expressed specific apprehensions about the removal of a historical landmark. He stated that “*[The] historic landmarks are being changed and that speaks to the soul of the community*.”

The Watts neighborhood holds a rich history that is deeply intertwined with the community’s identity. As such, the acquisition of land and redevelopment by housing corporations could disrupt the place attachment and sense of belonging of residents. Another stakeholder shared the following sentiment:

[Representative(s)] sat in front of me, and a few other people. And basically offered to consider selling back a sacred piece of land to the community, but then changed [their] mind. And [they’re] going forward with a plan to build on it the [new] cultural Crescent, which is, it’s like, if you can imagine seeing housing development built around the Brea Tar Pits.^1^ Or maybe even a better example, is high-rise housing in Leimert Park.^2^ It’s a cultural center. And [they’ve] through some political mechanisms, [they’ve] acquired the property, and now [they] wants to put more high-density housing in it.

In addition to the potential removal of important landmarks that cultivate connection to physical space and a sense of belonging, stakeholders also discussed broader changes to the built environment, which they argued impacts a sense of belonging.

You can see the transformation happening. But it’s happening around the folks that have lived here for a long time. So for instance, Freedom Plaza, that is a part of Jordan Downs redevelopment. All of those businesses that are coming in are great businesses, but you have a strip on 103rd street, with small businesses that have been there for years. And I don’t see that even though it was mentioned that there was going to be some assistance for those businesses. But the way I see it is that those businesses soon will be weeded out. They are going to be weeded out because everything is focused on those [new and] big stores now.

Stakeholders expressed concerns that these changes in the built and social environment could undermine community social cohesion, as residents may experience a diminished sense of belonging and both new and existing residents may face challenges with integration.

### 3.3. Residents’ Perceptions of Neighborhood Social Cohesion and Trust

Residents were surveyed to assess their levels of perceived community social cohesion and trust. Jordan Downs residents had considerably more favorable perceptions of their neighborhood relative to the comparison site residents. See Table 3. A significantly greater proportion of Jordan Downs residents strongly agreed or agreed (40%) that their neighborhood was a good place to live as opposed to the comparison sites (22%) (*p* < 0.001). Additionally, the overall social cohesion index score was significantly higher in Jordan Downs residents relative to the comparison site residents (difference of 0.33 standard deviation units; *p* < 0.001). When examining individual items within the Social Cohesion Scale, while all items suggested more favorable neighborhood perceptions about Jordan Downs residents, these differences relative to the comparison site residents were statistically significant for two items: a smaller proportion of Jordan Downs residents strongly agreed or agreed that people in their neighborhood do not get along (JD: 26%, comparison: 41%; *p* < 0.001) or do not share the same values (JD: 43%, comparison: 52%, *p* = 0.048) than comparison site residents. Next, we examined differences in neighborhood perceptions *within* Jordan Downs, namely between the residents of the new and old Jordan Downs units. Overall, 51% of New JD residents strongly agreed or agreed that their neighborhood was a good place to live, compared to only 35% in Old JD (*p* < 0.001). However, there were no statistically significant differences between New JD and Old JD residents in the overall social cohesion index score. With regard to the individual items on the Social Cohesion Scale, New JD residents had more favorable perceptions of some aspects (close-knit community, trust, and shared values), but had less favorable perceptions for other aspects (willingness to help neighbors, getting along with each other) relative to Old JD residents. None of these differences were statistically significant.

Perceived social cohesion was also analyzed by residential tenure, comparing residents who had lived at their respective site for 10 years or less versus more than 10 years (Table 4). No significant differences in social cohesion were observed for the overall social cohesion index score or the individual scale items, with the exception of neighborhood trust. A greater proportion of residents who had lived at their site for greater than 10 years endorsed the view that people in their neighborhood could be trusted compared to residents who had lived at their site for 10 years or less (≤10 years: 16%, >10 years: 26%; *p* = 0.008).

## 4. Discussion

Public housing redevelopment projects have potentially profound implications for social cohesion and its role in individual and community wellness. However, few studies have examined how public housing redevelopment in low-income neighborhoods is related to community social cohesion. The current study addresses this gap by conducting a mixed methods study to assess perceived social cohesion during a large public housing and neighborhood redevelopment. A unique feature of the redevelopment being studied is that residents were able to continue living in their homes while their neighborhood was being redeveloped. This created a valuable opportunity to study perceptions of social cohesion and belongingness during a large-scale redevelopment, considering perspectives from both stakeholders and residents. We assessed how community stakeholders perceived social cohesion through qualitative interviews. Quantitative survey data were then utilized to evaluate the extent to which these perceptions aligned with residents’ experiences of social cohesion. The use of qualitative and quantitative methods allows for a comprehensive understanding of social cohesion in the community.

Through the semi-structured interviews, stakeholders highlighted how the integration of existing and incoming residents, changes in the built environment, and opportunities for social interaction may impact social cohesion during redevelopment. Stakeholders expressed concerns about integrating new and existing Jordan Downs residents and its effects on the community, echoing longstanding critiques of housing and neighborhood redevelopment, particularly regarding gentrification [31,32]. These concerns reflect broader trends in urban redevelopment, where displacement and community fragmentation are common challenges [33]. Research on the HOPE VI Program shows that public housing redevelopment significantly alters the racial and economic composition of redeveloped neighborhoods [32]. While redevelopment advocates propose that this ‘social mixing’ of diverse groups through mixed-income housing serves to deconcentrate poverty and increase the social capital of minoritized residents, critics argue that the influx of new, often more affluent residents displaces current residents and disrupts existing community social networks [34–36]. In fact, the extant literature demonstrates that public housing redevelopment projects often result in displacement and do not generally foster positive social integration among current and new residents [31,37–40]. The perception that the integration of both current and new residents threatens the community’s existing social cohesion reflects discussions about the impact of diversity and mutual tolerance on social cohesion [6,41]. As discussed by Ahmadi [41], diversity alone does not threaten or cultivate social cohesion; rather, structural issues such as poverty, classism, and racism must be considered in relation to social cohesion. The concerns raised by participants regarding racial tension, resident integration, and gentrification support this, indicating that ‘social mixing’ alone is inadequate to promote community social cohesion.

The concerns around surveillance reported by stakeholders in this study have been reflected in other communities where redevelopment and gentrification have occurred [42,43]. In recognition of these displacement concerns, a strength of the Jordan Downs redevelopment is its ‘build first’ strategy in which new housing is built for current residents prior to the removal of older buildings to minimize physical displacement. However, as shared by interviewed stakeholders, the concerns with gentrification-induced displacements extend beyond just physical displacement and can also encompass the loss of culture, longstanding small businesses, social network ties, and a sense of community belonging. Displacement is not simply a loss of occupied physical space; it is also the loss of a neighborhood’s social character.

Yet, despite concerns raised in qualitative interviews with community stakeholders, our quantitative findings reveal a different narrative. Contrary to stakeholder perceptions and our initial hypothesis, Jordan Downs residents reported more positive views of their neighborhood during redevelopment, especially their relationships and shared values with neighbors. Differences in neighborhood perceptions between surveyed residents of the Old JD and New JD units were less consistent. Compared to residents in Old JD units, a greater proportion of residents in the New JD units endorsed the view that their neighborhood was a good place to live, although the social cohesion items were rated higher for some items but lower for others, yielding statistically insignificant differences in social cohesion between these groups. This suggests that while redevelopment may bring some positive perceptions, it could also have short-term destabilizing effects, as residents navigate changes in their environment, social networks, and community dynamics. These disruptions may hinder the full realization of community cohesion during or shortly after redevelopment.

In addition, the quantitative results indicate that resident tenure is associated with perceptions of one aspect of social cohesion, specifically trust in neighbors, but not others. Consistent with our hypothesis, residents who had lived in their respective neighborhood longer (>10 years) were more likely to trust people in their neighborhood compared to those with shorter tenures (≤10 years). This finding underscores the potential influence of neighborhood stability in terms of fostering trust and community connections.

The findings from qualitative interviews with stakeholders and survey responses from residents highlight their respective positionality within the Watts community. More specifically, public housing redevelopment carries a considerable degree of long-held stigma, often centered around fears of displacement, gentrification, and neighborhood change [32]. Given that the majority of community stakeholders in this study did not currently live in Watts (*n* = 15, 71%) and were highly educated (master’s or doctoral degree: *n* = 13, 62%), it is possible that their concerns around the Jordan Downs redevelopment were informed to a greater extent by general negative discourse towards redevelopment as opposed to lived experience within Jordan Downs.

The greater social cohesion perceived by Jordan Downs residents compared to the comparison site residents also challenges the traditional view linking public housing redevelopment to social fragmentation. Previous research on a gentrifying neighborhood by Buffel and Phillipson [17] observed that despite significant community changes, neighborhood networks endured through the use of communal gathering spaces and active social action engagements. From this perspective, public housing redevelopments may allow for opportunities for increased interactions between neighbors and community redefinition, ultimately contributing to increased social cohesion. It is possible that the greater social cohesion reported by Jordan Downs’ residents in contrast to residents of comparison sites is the result of resilience, adaptation, and redefinition in the face of neighborhood and housing redevelopment.

Another important consideration is that residents were allowed to remain in the community during redevelopment, and the early phases saw very few new residents moving in (and those were all low-income tenants) with no evidence of gentrification. The majority of new neighbors, including mixed-income tenants, are expected to join in the later phases. The relatively fewer changes to place features in these early stages of redevelopment may help to preserve identity and social cohesion among residents. Furthermore, the redevelopment had a strong community engagement component that extensively involved JD residents throughout the planning and implementation process with numerous opportunities for information sharing and feedback, which may have contributed to an increased sense of community and ownership. This community engagement may have also fostered institutional trust among residents, which has been implicated as a contributor to social cohesion [6,9]. Future work should assess how social cohesion evolves over time, particularly as the built environment and socioeconomic composition of residents is altered over the course of redevelopment.

### 4.1. Limitations

The study’s findings warrant careful interpretation in light of its inherent limitations. First, it is essential to acknowledge that this study used a purposive sample for the qualitative component, which can lead to under- or over-representation of the population’s perspectives regarding the Jordan Downs redevelopment. Consequently, the findings may carry a certain degree of self-selection bias stemming from the motivations that drove some community members to participate in the study, while others declined to be involved. Second, our study is cross-sectional and is therefore unable to assess whether and how neighborhood perceptions change over time, which should be pursued in future work. Another limitation lies in its specific geographic and cultural context. The majority of community members (in both the qualitative and quantitative studies) identify as Black/African American or Latino/a, and they all reside in Los Angeles County. Although the themes and experiences may hold relevance and transferability to other samples with similar characteristics [44], they can also hold geographic and selection biases, which limits the generalizability of the findings to other settings. Also, the sample of interviewed community stakeholders was not representative of the surveyed residents (e.g., varied levels of education). While this likely contributes to the differences in social cohesion findings between the groups, our two study populations provided valuable multi-level perspectives on the Jordan Downs redevelopment.

### 4.2. Conclusions

Redeveloping public housing communities and their surrounding neighborhoods is an important means of providing improved housing and neighborhood environments for socioeconomically disadvantaged individuals. However, its potential impact on existing community and social dynamics must also be considered. The current study indicates that despite the concerns of community stakeholders, residents had favorable perceptions of social cohesion during the Jordan Downs redevelopment. The concerns raised by various community stakeholders, particularly those centered on the integration of current and incoming residents, reveal the need for efforts to promote positive interactions among residents. Housing advocates and policymakers should additionally consider implementing methods to preserve longstanding spaces, landmarks, and businesses that are integral to residents’ place identity and the community fabric. This could include increasing investments in small businesses and establishing zoning regulations to protect historic or culturally significant locations.

Future research on redevelopment projects should examine additional factors that were beyond the scope of the present study. In particular, attention should be given to factors associated with social cohesion, such as community resilience [45], social capital [46], and land use diversity [47]. Additionally, while our quantitative survey primarily measured social cohesion between residents—or “micro-level” interactions—future research could also assess social cohesion between residents and groups (mezzo), and between residents and institutions (macro) [6,48]. Lastly, our findings suggest the importance of collecting longitudinal measures of social cohesion in communities through the life cycle of redevelopment. This is important given that some potential impacts of redevelopment (e.g., loss of cultural spaces) may occur over extended periods, leading to fluctuations in social cohesion over time.

## Figures and Tables

**Table 1.** Descriptive characteristics of Watts community stakeholders who completed semi-structured qualitative interviews (*n* = 21).

	% (*n*) or μ	SD; Range
*Gender*		
Female	62% (13)	
Male	38% (8)	
*Age (in years)*	47.3	13.5; 28–77
*Race/Ethnicity* ^a^		
Latino/a	43% (9)	
Black or African American	38% (8)	
Multiracial	10% (2)	
White	5% (1)	
Asian American and Pacific Islander	5% (1)	
*Highest Level of Education* ^b^		
High School degree	10% (2)	
Some college or Associates in Arts degree	5% (1)	
Bachelor’s degree	20% (4)	
Master’s degree	52% (11)	
Doctorate degree	10% (2)	
*Currently Lives in Watts*	29% (6)	
Tenure as a Watts resident (in years)	38.5	17.4; 16–68
*Organization Sector* ^c^		
Non-profit	57% (12)	
Education	38% (8)	
Housing Authority	33% (7)	
Government	33% (7)	
Employment	29% (6)	
Resident Leader	24% (5)	
Business	19% (4)	
Faith-based	14% (3)	
Law Enforcement	10% (2)	
Environmental	10% (2)	
Community Health	5% (1)	
*Tenure at Current Organization (in years)*	8.2	8.2; 1–30

*Notes*. μ = mean;

a multiracial individuals identified as Black/White and Latinx/Asian American;

b one community member did not answer this question;

c some participants selected more than one, therefore the total is greater than 100%; SD = standard deviation.

**Table 2.** Descriptive Characteristics of Watts Residents Who Completed Surveys (*N* = 647).

	Jordan Downs		Comparison Sites (*n* = 209)
	New JD (*n* = 131)	Old JD (*n* = 307)	
	*n* (%)	*n* (%)	*n* (%)
*Gender*			
Female	102 (78%)	224 (73%)	157 (75%)
Male	29 (22%)	83 (27%)	52 (25%)
*Age (in years)*			
18 to 34	52 (40%)	117 (38%)	79 (38%)
35 to 54	54 (41%)	114 (37%)	90 (43%)
55+	25 (19%)	76 (25%)	40 (19%)
*Ethnoracial Background*			
Latino/a	98 (75%)	240 (78%)	123 (59%)
Black/African American	33 (25%)	67 (22%)	86 (41%)
*Married*			
No	100 (76%)	233 (76%)	165 (79%)
Yes	31 (24%)	74 (24%)	44 (21%)
*Highest Level of Education*			
Less than High School	42 (32%)	89 (29%)	52 (25%)
High School or equivalent	50 (38%)	129 (42%)	84 (40%)
More than High School	39 (30%)	89 (29%)	73 (35%)
*Household Income*			
USD 0–USD 9999	49 (37%)	98 (32%)	101 (48%)
USD 10,000–USD 19,999	29 (22%)	83 (27%)	54 (26%)
USD 20,000+	53 (40%)	126 (41%)	54 (26%)

**Table 3.** Percentage who strongly agree or agree with statements about their neighborhood by location.

	Comparison (*n* = 209)	Jordan Downs (*n* = 438)	*p*-Value (JD vs. Comparison)	Old JD (*n* = 307)	New JD (*n* = 131)	*p*-Value (JD New vs. JD Old)
My neighborhood is a good place to live	22%	40%	**<0.001**	35%	51%	**0.001**
Social Cohesion Index Score, mean (SD)	2.8 (0.6)	3.0 (0.6)	**<0.001**	3.0 (0.6)	3.0 (0.6)	0.303
Social Cohesion Scale Items						
People around my neighborhood are willing to help their neighbors	45%	50%	0.156	51%	49%	0.661
My neighborhood is a close-knit community	33%	37%	0.352	35%	41%	0.206
People in my neighborhood can be trusted	19%	25%	0.104	24%	27%	0.562
People in my neighborhood generally don’t get along with each other^a^	41%	26%	**<0.001**	24%	29%	0.281
People in my neighborhood do not share the same values^a^	52%	43%	**0.048**	45%	40%	0.309

Notes:

a these two items were negative statements about the neighborhood and were reverse-coded to create the social cohesion index score.

**Table 4.** Percentage who strongly agree or agree with statements about their neighborhood by tenure at site.

	Tenure at Site		
	≤10 Years (*n* = 191)	>10 Years (*n* = 455)	*p*-Value of Diff by Tenure
My neighborhood is a good place to live	31%	35%	0.295
Social Cohesion Scale Items			
People around my neighborhood are willing to help their neighbors	47%	50%	0.508
My neighborhood is a close-knit community	35%	36%	0.857
People in my neighborhood can be trusted	16%	26%	0.008
People in my neighborhood generally don’t get along with each other	30%	31%	0.963
People in my neighborhood do not share the same values	48%	45%	0.469
Social Cohesion Index Score, mean (SD)	2.9 (0.6)	3.0 (0.6)	0.223
Sample: Adults in W4 (2021–2022), N = 646			

## Data Availability

The data that support the findings of this study are not publicly available, but the materials used in this study can be available from the Principal Investigator (last author) upon reasonable request, with appropriate ethical considerations.

## Footnotes

1 The La Brea Tar Pits is a group of tar pits known for preserving an extensive collection of Ice Age fossils. They are located in Los Angeles’ Miracle Mile, a cultural hub characterized by multiple museums, shopping centers, and historic architecture.

2 Developed in the 1920s, Leimert Park is a neighborhood located in South Los Angeles. Leimert Park is widely considered the focal point for Black culture, music, and art in Los Angeles.
